# Depressive Symptoms in Patients With SARS-CoV-2 and Acute Coronary Syndrome

**DOI:** 10.7759/cureus.103172

**Published:** 2026-02-07

**Authors:** Ana Gonjilashvili, Sophio Tatishvili

**Affiliations:** 1 Cardiology, New Vision University, Tbilisi, GEO; 2 Medicine, New Vision University, Tbilisi, GEO

**Keywords:** acs, assessment, depression, hyperinflammation, sars-cov-2

## Abstract

In 2020, the World Health Organization (WHO) declared the outbreak of Coronavirus disease 19 (COVID-19) caused by severe acute respiratory syndrome coronavirus 2 (SARS-CoV-2). Besides well-known pulmonary complications, other frequent detrimental health consequences included cardiovascular, thromboembolic, and mental health disorders. A high incidence of acute coronary syndromes (ACSs) with both short- and long-term outcomes was influenced by the disease severity and related complications, including mental health challenges, depression, and anxiety.

The aim of our study was to analyze the prevalence of depressive symptoms (somatic and cognitive) in patients with SARS-CoV-2 infection and concomitant cardiovascular disease (CVD).

Depression screening was conducted in 101 patients who survived SARS-CoV-2 infection during the 2020-2022 period. Of the study sample, 54 (53.5%) were female, and 47 (46.5%) were male, with the mean age of 55 (15.9 SD) years. All participants completed the Beck Depression Inventory (BDI) one to three months after hospital discharge. Patients with SARS-CoV-2 and ACS demonstrated a higher frequency of depressive symptoms compared to COVID-19-only patients without ACS. Statistically significant differences were observed in somatic symptoms (sleeplessness, irritability, loss of appetite, difficulties in concentration, tiredness), as well as in cognitive symptoms (loneliness, sadness, crying, fatigue, self-hatred, loss of interests, devaluation, indecision, and agitation).

Our study findings suggest that acute severe illness (SARS-CoV-2 and ACS), elderly age, and the presence of chronic diseases can contribute to depressive mood. These observations highlight the need for multidisciplinary care involving cardiologists, neurologists, psychiatrists, and rehabilitation specialists with a comprehensive assessment of depressive symptoms and consideration of classical risk factors, which can potentially affect prognosis. However, these findings should be interpreted as associative, given the cross-sectional design of the study and the presence of confounding factors.

## Introduction

Depression is common among patients with acute coronary syndrome (ACS) [1]. Evidence from recent papers demonstrates that new-onset depression was associated with increased risk of mortality in post-ACS patients compared to non-depressed ones [2]. It has been reported that people with cardiovascular disease (CVD) have elevated levels of depression and anxiety, with an occurrence ranging from 20% to 45% [3].

In 2020, the World Health Organization (WHO) declared a pandemic due to the outbreak of the coronavirus disease 19 (COVID-19, caused by SARS-CoV-2) [4,5]. In addition to pneumonia [the most common manifestation of the SARS-CoV-2 infection], frequent complications of SARS-CoV-2 infection included cardiovascular, thrombotic, and mental health disorders [6-8]. Cohort studies demonstrated a high incidence of ST-segment elevation myocardial infarction (STEMI) in SARS-CoV-2 infection patients [9]. The short- and long-term outcomes of SARS-CoV-2-related cardiovascular diseases are determined by the disease severity and related conditions and complications, including mental health challenges such as stress, depression, and anxiety, which have a detrimental effect [8]. According to a Chinese Cross-sectional study, the 10-year coronary heart disease risk was higher in post-SARS-CoV-2 patients with major depressive disorder (MDD) and higher systemic immune-inflammatory index (SII) [10].

The relationship between SARS-CoV-2 infection, cardiovascular diseases (CVD), and depression can be explained not only by biological mechanisms (hyperinflammation) but also by various social factors, such as an unhealthy lifestyle, social restrictions, and confinement [8,11].

Patients with long COVID syndrome and concomitant CVD may have shared symptoms such as: orthostatic hypotension, postural orthostatic tachycardia syndrome (POTS), syncope, chest pain, dyspnea, palpitations, chronic fatigue syndrome, 'brain fog', memory, cognitive and sleep difficulties, signs of impaired cardiac and cerebral perfusion, depression, and anxiety [12-14]. The identification of these symptoms requires a comprehensive approach.

Less attention is paid to the assessment of somatic and cognitive symptoms of depression in post-ACS patients. The aim of our observational study was to analyze the prevalence of depressive symptoms (somatic and cognitive) in patients with SARS-CoV-2 infection and concomitant CVD.

## Materials and methods

Participants 

Depression screening was conducted in 101 patients who survived severe acute respiratory syndrome Coronavirus 2 (SARS-CoV-2) infection during the 2020-2022 period. 

The study sample was recruited from different hospitals located in Tbilisi, Georgia. Patients were interviewed through telephone interviews and face-to-face contacts in hospital settings. Supplementary information (clinical data) was also obtained from the hospital's electronic medical record system. Before data collection, participants were provided a written or verbal consent form during outpatient visits or via telephone, with the standard consent form read aloud when applicable.

Depressive symptoms were assessed using a Beck Depression Inventory (BDI) within the three months after hospital discharge, to allow for clinical stabilization after the acute illness and to enable assessment of depressive symptoms during the post-acute phase. After assessment of depressive symptoms, the BDI score was categorized into two categories: >10 and <10. Our decision was based on the BDI interpreting manual, which concerns scores <10 as normal, 11-16 as mild mood disturbance, 17-20 as borderline clinical depression, 21-30 as moderate depression, 31-40 as severe depression, and >40 as extreme depression [15]. A cut-off value of >10 was based on the published literature stating a good sensitivity and specificity for outpatients, including the elderly [15].

Predictive variables and covariates

Fifty percent of SARS-CoV-2 positive patients were diagnosed with acute coronary syndrome (ACS), based on having chest pain, ST segment and T wave changes on electrocardiography (ECG), and positive troponin test [16]. Cardiac systolic and diastolic functions were assessed by echocardiography. Other covariates included arterial hypertension (AH) and diabetes mellitus (DM). 

To account for less frequent but clinically relevant comorbid conditions, we have built a composite variable for these diseases with two categories: 0/1. The category 1 included all other diseases that were present in different patients (such as thyroid disease, lymphoma, and colon cancer).

Statistical analysis

Descriptive statistical tests were used for the calculation of means and frequencies. A Chi-squared test was used for the estimation of the difference between groups. All tests were performed using the Statistical Package for the Social Sciences (SPSS; IBM Inc., Armonk, New York). The p-value threshold was set as 0.005 with 95% confidence intervals.

## Results

Out of 101 SARS-CoV-2 positive patients, 54 (53.5%) were females and 47 (46.5%) males. The mean age was 55 (15.9 SD) years. The mean depression score was 15.4 (9.2 SD). Fifty percent of SARS-CoV-2-positive patients suffered from concomitant ACS. Patients with SARS-CoV-2 and acute coronary syndrome were older and had more cardiometabolic risk factors, such as arterial hypertension, diabetes mellitus, and previous coronary disease. Other clinical characteristics are given in Table 1.

**Table 1:** General characteristics of the sample (N=101) according to SARS-CoV-2 status and acute coronary syndrome Notes: Sample size 101 patients. Results expressed by mean and standard deviation or frequencies with percentages (n, %). Chi-squared tests for contingency tables were applied and independent samples T-test was used to compare means. The p-value was set at 0.05 ACS - acute coronary syndrome; CAD - coronary artery disease

N=101	Total n (%)	SARS-CoV-2 with ACS, n=51	SARS-CoV-2 without ACS, n=50	Pearson Chi-squared (χ²) test	Degrees of freedom	Gramer's V test - effect size	p-value
Age (years)	55 (15.9)	64 (12)	46.4 (14.5)	-	-	-	<0.001
Males	47 (46.5%)	30 (63.8%)	17 (36.2%)	6.25	1	0.249	0.012
Females	54 (53.5%)	21 (38.9%)	33 (61.1%)	6.25	1	0.249	0.012
Depression score	15.4 (9.2)	21.8 (6.9)	8.8 (6.0)	-	-	-	<0.001
Arterial hypertension	71 (70.3%)	50 (70.4%)	21 (29.6%)	37.9	1	0.613	<0.001
Diabetes mellitus	19 (18.8%)	15 (78.9%)	4 (21.1%)	7.58	1	0.274	0.005
Ejection fraction (mean)	50 (8.6)	45.4 (8.3)	54.6 (6.2)	-	-	-	<0.001
Previous CAD	29 (28.7%)	24 (82.8%)	5 (17.2%)	17.5	1	0.419	<0.001
Other diseases	22 (21.8)	13 (59.1%)	9 (40.9%)	0.831	1	0.091	0.362

Assessment of depressive symptoms

The mean age of patients with a higher depressive score (>10 points) was 62 years. Seventy-four percent of males and 64% of females had a higher number of depressive symptoms (more than 10 points). A higher depressive score (>10 points) was observed in 50 (71.4%) patients from SARS-CoV-2 positive patients with acute coronary syndrome, compared to 20 (28.6%) patients with only SARS-CoV-2. Likewise, depression score >10 was observed in elderly patients as well as in those with arterial hypertension and mildly reduced ejection fraction, indicating that advanced age and cardiometabolic risk factors could be considered as potential confounding factors (Table 2).

**Table 2:** Difference between groups for depressive scores Notes: Sample size 101 patients. Results expressed by mean and standard deviation or frequencies with percentages (n, %). Chi-squared tests for contingency tables were applied and independent samples T-test was used to compare means. The p-value was set at 0.05 ACS - acute coronary syndrome; CAD - coronary artery disease

N=101	Total n (%)	Depressive symptoms <10	Depressive symptoms >10	Pearson Chi-squared (χ²) test	Degrees of freedom	Gramer's V test - effect size	p-value
Age	-	38.4 (9.0)	62.5 (12.3)	-	-	-	<0.001
Covid and ACS	51 (50.5%)	1 (2%)	50 (98%)	39.9	1	0.629	<0.001
COVID without ACS	50 (49.5%)	30 (60%)	20 (40%)	39.9	1	0.629	<0.001
Males	47 (46.5%)	12 (25 %)	35 (74%)	1.10	1	0.104	0.294
Females	54 (53.5%)	19 (35.2%)	35 (64.8%)	1.10	1	0.104	0.177
Arterial hypertension	71 (70.3%)	6 (8.5%)	65 (91.5%)	55.6	1	0.742	< 0.001
Diabetes mellitus	19 (18.8%)	0 (0%)	19 (100%)	10.4	1	0.320	0.001
Previous CAD	29 (29%)	0 (0%)	29 (100%)	18.3	1	0.428	<0.001
No previous history of CAD	71 (71%)	31 (43.7%)	40 (56.3%)	18.3	1	0.428	<0.001
Ejection fraction	50 (8.6)	55 (4.8)	47 (8.7)	-	-	-	<0.001
Other diseases	22 (21.8%)	3 (13.6%)	19 (86.4%)	3.84	1	0.195	0.050

Covid and acute coronary syndrome (ACS) patients tended to present a higher frequency of depressive symptoms compared to those without acute coronary syndrome (ACS). Statistically Significant differences were observed in somatic (sleeplessness, irritability, loss of appetite, difficulties in concentration, tiredness), as well as in cognitive symptoms (loneliness, sadness, crying, fatigue, self-hatred, loss of interests, devaluation, indecision, and agitation). A stronger association was found between SARS-CoV-2 patients with acute coronary syndrome (ACS) and somatic symptoms of depression (Table 3).

**Table 3:** Difference between groups in depressive symptoms Notes: Sample size 101 patients. Results expressed by frequencies with percentages (n, %). Chi-squared tests for contingency tables were applied, and the p-value was set at 0.05. ACS - acute coronary syndrome

N=101	Total n (%)	COVID and ACS	COVID without ACS	Pearson Chi-squared (χ²) test	Degrees of freedom	Gramer’s V test - effect size	p-value
Cognitive symptoms							
Sadness	75 (74.3%)	48 (64%)	27 (36%)	21.3	1	0.459	< 0.001
Loss of pleasure	65 (64.4%)	43 (66.2%)	22 (33.8%)	17.89	1	0.421	< 0.001
Difficulty with oneself	50 (49.5%)	35 (70%)	15 (30%)	15.1	1	0.386	< 0.001
Self criticism	54 (53.5%)	36 (66.7%)	18 (33.3%)	12.1	1	0.347	< 0.001
Crying	43 (42.6%)	27 (62.8%)	16 (37.2%)	4.53	1	0.212	0.033
Agitation	46 (45.5%)	28 (61%)	18 (39.1%)	3.64	1	0.190	0.057
Loss of interests	45 (44.6%)	31 (68.9%)	14 (31.1%)	10.98	1	0.330	0.001
Indecision	52 (51.5%)	36 (69.2%)	16 (30.8%)	15.0	1	0.366	< 0.001
Devaluation	60 (59.4%)	42 (70%)	18 (30%)	22.49	1	0.472	< 0.001
Somatic symptoms							
Sleeplessness	71 (70.3%)	48 (67.6%)	23 (32.4%)	27.9	1	0.526	< 0.001
Irritability	72 (71.3%)	49 (68.1%)	23 (31.9%)	30.9	1	0.553	< 0.001
Loss of appetite	57 (56.4%)	43 (75.4%)	14 (24.6%)	32.56	1	0.568	< 0.001
Difficulties in concentration	57 (56.4%)	45 (78.9%)	12 (21.1%)	42.37	1	0.648	< 0.001
Tiredness	80 (79.2%)	49 (61.3%)	31 (38.8%)	17.80	1	0.420	< 0.001
Sex interests	69 (68.3%)	49 (71%)	20 (29%)	36.68	1	0.603	< 0.001

## Discussion

Main findings from our observational study suggest that post-acute coronary syndrome (ACS) patients with COVID-19 infection have a higher prevalence of cognitive (loneliness, sadness, crying, self-hatred, loss of interests, devaluation, indecision, and agitation) and somatic (sleeplessness, irritability, loss of appetite, difficulties in concentration, tiredness) symptoms of depression. These findings are in line with other studies demonstrating an increased prevalence of depressive symptoms (31%) in post COVID patients [17] It was proposed that direct myocardial and neuroinvasion of the virus through angiotensin-converting enzyme 2 (ACE2) receptors [18], hyperinflammation, unhealthy lifestyle, social restrictions and non-coding microRNAs (miRNAs) may play a major role in pathogenesis of non-communicable diseases (acute coronary syndrome, depression) and could contribute to brain-heart cross-talk in SARS-CoV-2 patients [19,20].

Immune-mediated cytokine storm promotes hypercoagulation, micro and macroangiopathy, and vascular thrombosis via platelet, toll-like receptors (TLRs), and macrophages activation [21-23]. Macrophage-induced disruption of atherosclerotic plaques results in myocardial infarction [21]. The virus can reach brain microvessels, leading to endothelial dysfunction and microthrombotic events [20].

Along with systemic inflammation, brain-heart communication relies on extracellular vesicles (EVs), which carry biological information and can facilitate inter-organ communication between heart and brain in patients with SARS-CoV-2 infection [24]. High levels of EVs were related to increased coagulation and thrombotic events [25].

SARS-CoV-2-related cytokine storm may promote the conversion of tryptophan into kynurenine and hyperactivation of the kynurenine pathway, and its toxic metabolites (quinolinic acid, 30-hydroxykynurenine, and 3-hydroxy-anthranilic acid), leading to decreased serotonin synthesis and therefore depressive disorder [26,27]. It was reported that the development of acute myocardial infarction was attributed to overactivation of the kynurenine pathway in patients with stable angina [28]. Our results indicate that a higher number of depressive symptoms was observed in patients with previous coronary artery disease. The National Adult Cardiac Rehabilitation (NACR) registry reported that during pandemics, the risk of newly developed depression was high in patients with pre-existing coronary disease [29].

While not fully understood, it is considered that systemic inflammation and cytokine storm in SARS-CoV-2 may lead to autonomic dysfunction and related cardio-neuro symptoms, such as fatigue, sleep difficulties, anxiety, depression, etc. [12]. SARS-CoV-2-related dysautonomia - the imbalance of parasympathetic and sympathetic systems leads to impaired function of cardiovascular and neurological systems [13,30]. Neuroinflammation and related sleep loss could be considered as possible contributors to depressive symptoms in post-COVID patients [31]. In our sample, 67% of patients with ACS and COVID-19 reported sleeplessness during depression screening. Depression, anxiety, and insomnia negatively affected the coping process in heart failure patients during the SARS-CoV-2 pandemic [32].

Fatigue was reported in 61% of our patients with SARS-CoV-2 and ACS during depression screening. According to published data, fatigue is the most common symptom among patients with long COVID syndrome [33]. Fatigue may be a shared symptom observed in patients with post-COVID syndrome, depression, or ACS. Therefore, its assessment is very important to differentiate between cardiac and psychiatric conditions [12]. Persistive fatigue, ACS-related myocardial dysfunction, social restrictions, and isolation could contribute to neuropsychiatric manifestations [34]. In our study, we identified a higher number of cognitive symptoms of depression (sadness, loss of pleasure, loss of interest, etc.) in patients with ACS with SARS-CoV-2 infection. Higher prevalence of cognitive symptoms could be attributed to social limitations, which contributed to worsening of mental health status in patients with pre-existing cardiovascular diseases [35].

Increased state of systemic inflammation and presence of depression can predict high risk of coronary heart disease and therefore adverse prognosis [10]. Studies report decreased functional capacity and survival in heart failure patients after SARS-CoV-2 infection [36]. Elderly age, arterial hypertension, and diabetes mellitus are related to chronic low-grade inflammation and may contribute to depressive mood in these patients [37,38].

Therefore, assessment of depressive symptoms is crucial in post-ACS patients. In clinical practice, the presence of somatic symptoms of depression (fatigue, sleeplessness, loss of appetite) is mainly explained by the main disease, while recognition of depression is based on identification of cognitive symptoms of depression [39]. However, long-term prognosis is associated with somatic symptoms of depression [39]. Therefore, assessment of depressive symptoms is crucial in post-acute coronary syndrome patients. 

Our study has some limitations. First, the sample size was relatively small, which may limit the generalizability of the findings. 

Second, the ACS group was clinically heterogeneous, including only 50% of troponin-positive patients. Nevertheless, these patients were considered high risk, as all were admitted with diagnoses of acute cardiovascular events, the majority had previous CVD, and most had multiple CVD risk factors. All patients were clinically managed and treated as having ACS; therefore, we retained the term ACS for this group rather than introducing an alternative classification (e.g., cardiac disease severity).

Third, 22% of the total sample had comorbid conditions (such as thyroid disease, lymphoma, and colon cancer). Having chronic diseases could potentially contribute to the depressive mood due to chronic low-grade inflammation, potentially compounding the observed associations. 

Fourth, information on smoking status, obesity, and dyslipidemia was not available for every patient due to incomplete hospital records. Inclusion of these variables could have provided additional insight into the relationship between cardiovascular risk factors and depressive symptoms. Chronic inflammatory states related to hypertension and diabetes may also play a role in the development of depressive mood. However, the cross-sectional design of our study does not permit us to make conclusions about the causal relationship between SARS-CoV-2, comorbid conditions, and depression. Therefore, these findings should be interpreted with caution in the context of potential confounding factors and the hypothetical nature of the above-mentioned biological mechanisms.

Finally, depressive symptoms were assessed only once, within one to three months after SARS-CoV-2 infection. Consequently, we were unable to evaluate the trajectory of depressive symptoms or changes for long-term follow-up.

## Conclusions

Our study findings suggest that acute severe illness (SARS-CoV-2 and ACS), elderly age, and the presence of chronic diseases can contribute to depressive mood. These observations highlight the need for multidisciplinary care involving cardiologists, neurologists, psychiatrists, and rehabilitation specialists with a comprehensive assessment of depressive symptoms and consideration of classical risk factors, which can potentially affect prognosis. However, these findings should be interpreted as associative, given the cross-sectional design of the study and the presence of confounding factors.
